# miR‐485‐3p targets SIRT1 in vascular smooth muscle cells mediating the occurrence of aortic dissection

**DOI:** 10.1111/jcmm.18454

**Published:** 2024-07-15

**Authors:** Yuling Xie, Linfeng Xie, Zhihuang Qiu, Jian He, Fei Jiang, Meiling Cai, Yanjuan Lin, Liangwan Chen

**Affiliations:** ^1^ Department of Cardiovascular Surgery Fujian Medical University Union Hospital Fuzhou Fujian P. R. China; ^2^ Key Laboratory of Cardio‐Thoracic Surgery (Fujian Medical University) Fujian Province University Fuzhou Fujian P. R. China; ^3^ Department of Nursing Fujian Medical University Union Hospital Fuzhou Fujian P. R. China

**Keywords:** aortic dissection, HAVSMCs, miR‐485‐3p, SIRT1

## Abstract

Studies have demonstrated a close correlation between MicroRNA and the occurrence of aortic dissection (AD). However, the molecular mechanisms underlying this relationship have not been fully elucidated and further exploration is still required. In this study, we found that miR‐485‐3p was significantly upregulated in human aortic dissection tissues. Meanwhile, we constructed in vitro AD models in HAVSMCs, HAECs and HAFs and found that the expression of miR‐485‐3p was increased only in HAVSMCs. Overexpression or knockdown of miR‐485‐3p in HAVSMCs could regulate the expression of inflammatory cytokines IL1β, IL6, TNF‐α, and NLRP3, as well as the expression of apoptosis‐related proteins BAX/BCL2 and Cleaved caspase3/Caspase3. In the in vivo AD model, we have observed that miR‐485‐3p regulates vascular inflammation and apoptosis, thereby participating in the modulation of AD development in mice. Based on target gene prediction, we have validated that SIRT1 is a downstream target gene of miR‐485‐3p. Furthermore, by administering SIRT1 agonists and inhibitors to mice, we observed that the activation of SIRT1 alleviates vascular inflammation and apoptosis, subsequently reducing the incidence of AD. Additionally, functional reversal experiments revealed that overexpression of SIRT1 in HAVSMCs could reverse the cell inflammation and apoptosis mediated by miR‐485‐3p. Therefore, our research suggests that miR‐485‐3p can aggravate inflammation and apoptosis in vascular smooth muscle cells by suppressing the expression of SIRT1, thereby promoting the progression of aortic dissection.

## 1 INTRODUCTION

Aortic dissection (AD) is a life‐threatening medical emergency caused by the tearing of the inner layer of the aorta or bleeding within the aortic wall.^1^, ^2^ Research has shown that the annual incidence rate of acute AD is approximately three cases per 100,000 people, meanwhile, the mortality rate of this disease is extremely high, with a 1%–3% increase in mortality rate for every 1‐h delay.^3^ The exact mechanism of AD is still not fully understood. The most common causes of AD include intrinsic connective tissue disorders, arterial hypertension and atherosclerosis.^4^, ^5^ Currently, the clinical management of AD primarily relies on surgical intervention, and there are few effective drug treatment options available.^6^, ^7^ Therefore, it is crucial to conduct in‐depth research on the molecular mechanisms of AD and to identify new therapeutic targets.

MicroRNAs (miRNAs) are a class of non‐coding RNA molecules, with a length of approximately 20–24 nucleotides. They participate in the occurrence and development of various diseases by complementary pairing with target mRNA sequences.^8^ MiRNAs are crucial regulatory factors in AD. MiRNAs such as miRNA‐30a, miR‐145, miR222, etc., can participate in the regulation of AD development by modulating cellular inflammation, apoptosis, proliferation and other processes.^9^, ^10^, ^11^ miR‐485‐3p plays an important role in various diseases such as Alzheimer's disease, pancreatic cancer, and atherosclerosis, but its role in AD has not been reported in studies.^12^, ^13^, ^14^

Silencing information regulator 2 related enzyme 1 (SIRT1) is a highly conserved NAD‐dependent deacetylase, belonging to the sirtuin family.^15^ SIRT1, as a post‐translational regulator, plays a role in regulating various diseases including diabetes, cancer, cardiovascular diseases and neurodegenerative diseases.^16^, ^17^ Multiple studies have reported that SIRT1 expression is reduced in aortic dissection or aneurysm, and the upregulation of SIRT1 in endothelial cells or smooth muscle cells can block the occurrence of diseases by reducing cellular oxidative stress and inflammatory responses.^18^, ^19^

In this study, we observed an upregulated expression of miR‐485‐3p in the aortic tissues of AD patients and in Human Aortic Vascular Smooth Muscle Cells (HAVSMCs). Both in vivo and in vitro studies revealed that miR‐485‐3p plays a role in regulating inflammation and apoptosis in vascular smooth muscle cells, thereby contributing to the development of AD. Through molecular mechanism research, we identified that miR‐485‐3p negatively targets SIRT1. In vivo studies demonstrated that the activation of SIRT1 alleviates AD occurrence. Furthermore, functional rescue experiments showed that overexpression of SIRT1 can counteract the inflammation and apoptosis in HAVSMCs induced by miR‐485‐3p. Therefore, the aim of this study was to investigate whether miR‐485‐3p can regulate the inflammation and apoptosis of HAVSMCs by modulating the expression of SIRT1, thus influencing the development of AD and providing new targets for its treatment.

## 2 MATERIALS AND METHODS

### 2.1 Clinical sample collection

Aortic tissues from AD patients and healthy people were collected between August 2022 and June 2023 from the Union Hospital of Fujian Medical University (*N* = 12). In the group with AD, the studied aortic tissues were taken from the area within 1 cm of the rupture site in the ascending aorta. As a negative control, the same region of the aorta were harvested from healthy donors during organ transplantation procedures. The samples were immediately flash‐frozen in liquid nitrogen and stored at −80°C until further use. The clinical information of the subjects in the two groups were presented in Table S1. This research project was conducted following the principles outlined in the Declaration of Helsinki and approved by the Ethics Committee of the Union Hospital of Fujian Medical University.

### 2.2 Cell culture and treatment

HAVSMCs (CP‐H081), HAECs (CP‐H080), and HAFs (CP‐H219) were purchased from Procell (Wuhan, China). They were respectively cultured in smooth muscle cell culture medium (CM‐H081), endothelial cell culture medium (CM‐H080) and vascular fibroblast cell culture medium (CM‐H219), and were cultured in a humidified incubator at 37°C with 5% CO_2_. Cells from passages 3 to 5 were used for further experiments. HEK293 cells were purchased from ATCC (Manassas, VA, USA), and cultured in DMEM (Corning) supplemented with 10% FBS (Giboco) at 37°C and 5% CO2.

PDGF‐BB is an important mitogen that can promote the phenotype transformation and migration of HAVSMCs, and is a major factor in the development of AD. To simulate AD in vitro, HAVSMCs were exposed to 20 ng/mL of PDGF‐BB (Sigma, USA) for 48 h. To investigate the role of miR‐485‐3p in an in vitro simulated AD model, HAVSMCs were transfected with miR‐485‐3p mimic (50 nM), inhibitor (100 nM), or negative controls (RiboBio, Guangzhou, China) using Lipofectamine 2000 (Invitrogen). After 24 h of transfection, the cells were exposed to 20 ng/mL PDGF‐BB for 48 h. Meanwhile, SIRT1 overexpression or knockdown plasmids were transfected in HAVSMCs to investigate the function of target genes of miR‐485‐3p.

### 2.3 CCK‐8 Assay

Cell Counting Kit‐8 (Beyotime Biotechnology, C0037, Shanghai) was used to evaluate cell viability. At the end of the experiment, 10 μL of CCK‐8 solution was added to the cells and incubated for 2 h in the dark. Absorbance at 450 nm was measured using a microplate reader (Bio‐Rad).

### 2.4 Animals

AD model: 4‐week‐old male mice were fed a regular diet and administered BAPN (Sigma‐Aldrich, St. Louis, MO, USA) dissolved in drinking water at a dose of 1 g/kg per day for 4 weeks. At 8 weeks of age, the mice were anaesthetised with isoflurane inhalation (induction dose: 3%–5%, maintenance dose: 1%–2%; Harvey‐bio, Beijing, China), and osmotic minipumps (Alzet, Cupertino, CA, USA) filled with AngII (Sigma‐Aldrich, St. Louis, MO, USA) at a rate of 1 μg/kg per min were subcutaneously implanted.

AAV9 treatment: Mice were treated with AAV9 vectors carrying SM22α promoter‐driven miR‐485‐3p overexpression or miR‐485‐3p knockdown, along with their control gene (GeneChem, China), via tail vein injection at a dose of 10^11^ vg/mice.

EX‐527 treatment: Mice received EX‐527 (20 mg/kg) daily by gavage for 4 weeks.

SRT 1720 treatment: Mice were orally administered with SRT 1720 (50 mg/kg) daily for 4 weeks.

### 2.5 Haematoxylin and eosin staining

The aortic blood vessel tissues were fixed in 4% PFA and then embedded in paraffin. The paraffin sections were dewaxed and dehydrated. HE staining was completed using a Hematoxylin and Eosin Staining Kit (Keygen, China). Images were captured using a Nikon Eclipse microscope (Nikon, Japan) and analysed using NIS Elements software (Nikon).

### 2.6 Elastin Van Geison staining

The aortic blood vessel tissues were fixed in 4% PFA and cut into 5 μm sections. Elastin Van Geison staining was carried out using kit (Zhongshan Golden Bridge Biotechnology, Beijing, China) according to the provided protocol. Images were captured using a Nikon Eclipse microscope (Nikon, Japan) and analysed using NIS Elements software (Nikon).

### 2.7 Masson's trichrome staining

The aortic blood vessel tissues were fixed in 4% PFA and then embedded in paraffin. The 5‐μm‐thick sections were fixed with 4% PFA and then subjected to Masson's trichrome staining (Servicebio, China) according to the manufacturer's instructions. Images were captured using a Nikon Eclipse microscope (Nikon, Japan) and analysed using NIS Elements software (Nikon).

### 2.8 Real‐time PCR

Total RNA was isolated from tissues or cultured cells using Trizol RNAiso Plus (TaKaRa). The RNA concentration and quality were determined by NanoDrop (Thermo Scientific). A total of 400 ng RNA was then reverse transcribed to cDNA using a RevertAid First Strand cDNA synthesis kit (Thermo Scientific K1622). Real‐time PCR was conducted using TaKaRa SYBR Premix Ex Taq (Tli RNaseH Plus, Japan) on a Roche LightCycler 480 PCR system. The 2^−ΔΔCT^ method was used for data quantification. Primers for miR‐485‐3p and internal reference U6 were obtained from RiboBio (Guangzhou, China). The primer sequences for the regular gene are listed in Table S2.

### 2.9 Western blotting

Total protein was lysed from tissues and cells with RIPA lysis buffer (KeyGEN BioTECH, China) complemented with 1% phenylmethylsulfonyl fluoride (PMSF) and Pierce protease and phosphatase inhibitor (Thermo Scientific, 88668). Equal quantities of total protein were separated by Sodium Dodecyl Sulfate‐Polyacrylamide Gel Electrophoresis (SDS‐PAGE) and transferred onto Polyvinylidene Fluoride (PVDF) membranes. Protein blots were blocked with 5% skim milk and incubated with primary antibodies for Caspase3 (Proteintech, 66470‐2‐Ig), BAX (Abclonal, A0207), BCL2 (Abclonal, A2845), NLRP3 (Proteintech, 19771‐1‐AP), SIRT1 (Proteintech, 13161‐1‐AP), and β‐actin (Proteintech, 81115‐1‐RR) at 4°C overnight. Corresponding secondary antibodies were incubated on the next day before detection for protein bands using an enhanced chemiluminescence (ECL) kit. Finally, protein band intensity was analysed using ImageJ software.

### 2.10 Luciferase reporter assay

To investigate the direct interaction between miR‐485‐3p and the 3' UTR of SIRT1, we constructed a luciferase reporter vector, pGL3‐Basic (Promega), which contains binding sites (or mutant sites) with miR‐485‐3p in the 3' UTR of SIRT1. The wild‐type (WT) or mutant (mut) luciferase reporter plasmids were transfected into 293 T cells, along with miR‐485‐3p mimic or negative control. The dual‐luciferase reporter assay was performed according to the manufacturer's instructions (Promega).

### 2.11 Statistical Analysis

Prism 8.0 was used for data processing and analysis. All experiments were repeated three times. Data are presented as mean ± standard deviation. Student's *t*‐test was used for mean comparison between two groups. For multiple group comparisons, two‐way analysis of variance (anova) was performed followed by Tukey's post hoc test. A *p*‐value less than 0.05 was considered statistically significant.

## 3 RESULTS

### 3.1 High expression of miR‐485‐3p in human aortic dissection

**FIGURE 1:**
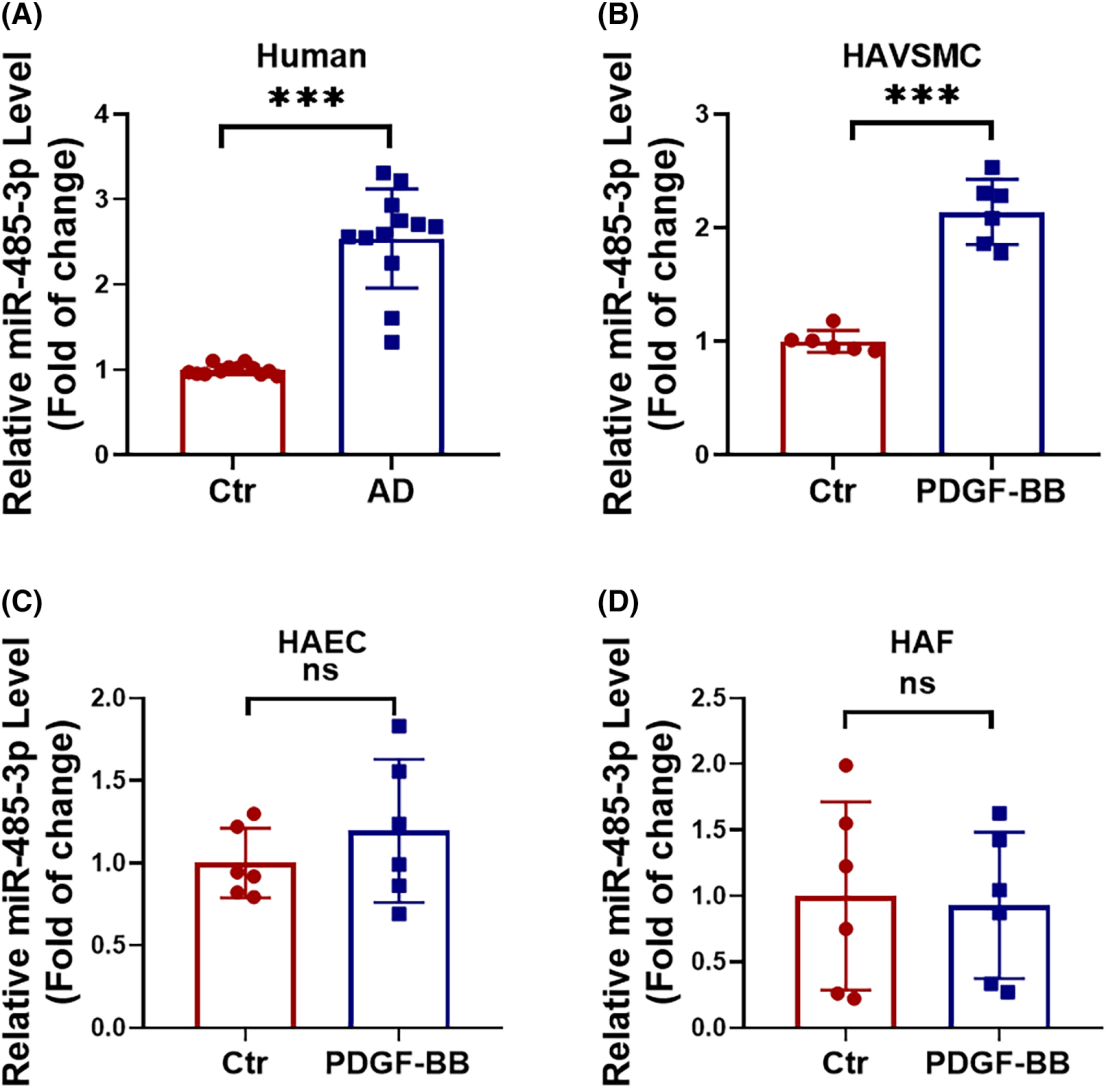
Increased expression of miR‐485‐3p in aortic dissection. (A–D) RT‐PCR was performed to assess the expression of miR‐485‐3p in a healthy control group and aortic dissection (AD) patient group (*N* = 12), as well as the expression of miR‐485‐3p in HAVSMCs, HAECs and HAFs treated with PDGF‐BB (*N* = 6). Data between two groups were compared by unpaired two‐tailed Student's *t*‐test. ***p < 0.001.

Several studies have already constructed miRNA expression profiles of AD using bioinformatics analysis, and they have reported an upregulation trend of miR‐485‐3p in AD patients.^20^, ^21^ RT‐PCR verification revealed that the expression of miR‐485‐3p was significantly increased in the aortic tissues of AD patients (Figure 1A). Meanwhile, we also observed a significant increase in inflammation response and apoptosis level in AD patients (Figure S1). To determine the specific aortic vascular cell type regulated by miR‐485‐3p, we extracted HAVSMCs, HAECs, and HAFs and treated them with PDGF‐BB to establish an in vitro AD model. The results demonstrated that miR‐485‐3p was highly expressed in the HAVSMCs of the model group (Figure 1B), but showed no significant difference in HAECs and HAFs (Figure 1C,D). Additionally, inflammation and apoptosis were significantly increased in the HAVSMCs of the model group (Figure S2). In conclusion, we found that miR‐485‐3p is upregulated in AD and may mediate inflammation and apoptosis in HAVSMCs.

### 3.2 miR‐485‐3p promotes inflammation and apoptosis in HAVSMCs

**FIGURE 2:**
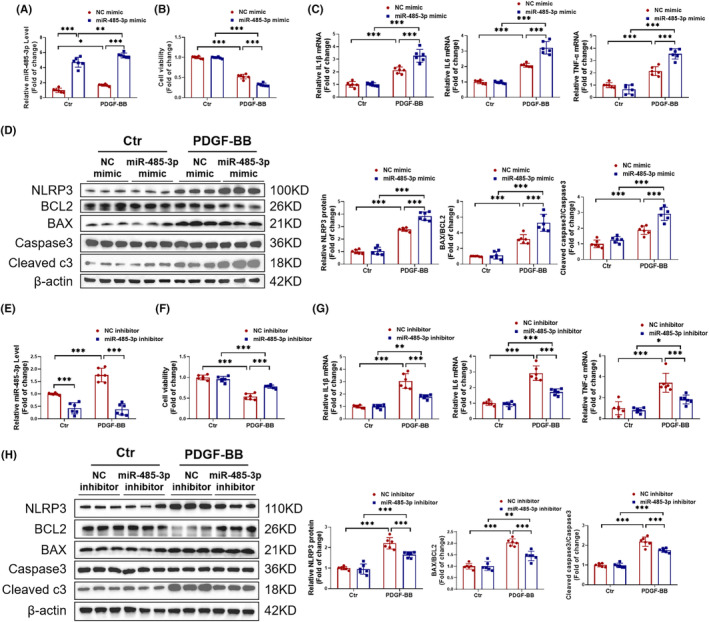
miR‐485‐3p promotes inflammation and apoptosis in Human Aortic Vascular Smooth Muscle Cells (HAVSMCs). (A–D) HAVSMCs were transfected with miR‐485‐3p mimic and treated with PDGF‐BB. (A) RT‐PCR confirmed the transfection efficiency of miR‐485‐3p mimic (*N* = 6). (B) CCK‐8 assay was used to evaluate the impact of miR‐485‐3p overexpression on the viability of HAVSMCs (*N* = 6). (C) RT‐PCR for the expression levels of inflammatory cytokines IL1β, IL6 and TNF‐α (*N* = 6). (D) Western blot to evaluate the protein expression levels of NLRP3, BAX/BCL2 and Cleaved caspase3/Caspase3 (*N* = 6). (E–H) HAVSMCs were transfected with miR‐485‐3p inhibitor and treated with PDGF‐BB. (E) RT‐PCR confirmed the transfection efficiency of miR‐485‐3p inhibitor (*N* = 6). (F) CCK8 assessed the effect of miR‐485‐3p knockdown on cell viability in HAVSMCs (*N* = 6). (G) RT‐PCR measured the expression of inflammatory cytokines IL1β, IL6, and TNF‐α (*N* = 6). (H) Western blot detected the expression of NLRP3, BAX/BCL2 and Cleaved caspase3/Caspase3 (*N* = 6). Data among four groups were compared by two‐way anova followed by Tukey's post hoc test. *p < 0.05, **p < 0.01, ***p < 0.001.

To investigate the relationship between miR‐485‐3p and HAVSMCs, we transfected miR‐485‐3p mimic and inhibitor into HAVSMCs and established an AD model. We observed that miR‐485‐3p overexpression further reduced the cell viability of HAVSMCs treated with PDGF‐BB (Figure 2A,B). Meanwhile, RT‐PCR revealed an increase in the expression of pro‐inflammatory cytokines IL1β, IL6 and TNF‐α (Figure 2C). Western blot also showed an increased expression of the inflammatory mediator NLRP3, as well as an increased ratio of the apoptotic proteins BAX/BCL2 and Cleaved caspase3/Caspase3 (Figure 2D). Similarly, the knockdown of miR‐485‐3p reversed the decrease in cell viability induced by PDGF‐BB in HAVSMCs (Figure 2E,F). Additionally, the RT‐PCR results showed that knocking down miR‐485‐3p reduced the expression of pro‐inflammatory cytokines IL1β, IL6 and TNF‐α in HAVSMCs (Figure 2G). Western blot analysis also revealed an increased expression of the inflammatory mediator NLRP3, as well as an increased ratio of apoptotic proteins BAX/BCL2 and Cleaved caspase3/Caspase3 (Figure 2H). In addition, we found that miR‐485‐3p does not regulate the proliferation, migration, and phenotypic transformation of HAVSMCs (Figure S3). Overall, miR‐485‐3p is involved in regulating inflammation and apoptosis in HAVSMCs.

### 3.3 VSMC‐specific overexpression of miR‐485‐3p promotes the development of AD in mice

**FIGURE 3:**
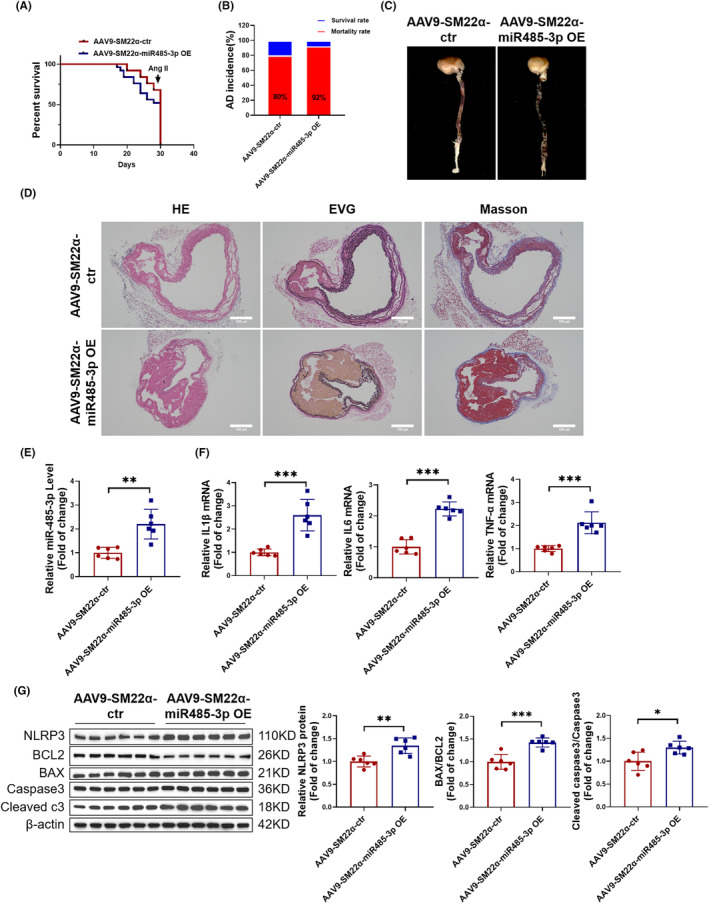
VSMC‐specific overexpression of miR‐485‐3p promotes the development of aortic dissection (AD) in mice. (A) Mouse survival curve treated with AAV9‐SM22α‐miR‐485‐3p OE or AAV9‐SM22α‐ctr followed by AD treatment (*N* = 25). (B) The incidence of AD in mice treated with AAV9‐SM22α‐miR‐485‐3p OE (*N* = 25). (C) Images of mouse aortic vascular tissue treated with AAV9‐SM22α‐miR‐485‐3p OE. (D) Observation of aortic wall and elastic fibre arrangement in AD mice treated with AAV9‐SM22α‐miR‐485‐3p OE using HE staining, EVG staining, and Masson staining, scale bar = 100 μm (*N* = 5). (E) Detection of miR‐485‐3p expression in AD mouse aortas treated with AAV9‐SM22α‐miR‐485‐3p OE by RT‐PCR (*N* = 6). (F) mRNA expression levels of inflammatory factors IL1β, IL6 and TNF‐α in AD mouse aortic vascular tissue treated with AAV9‐SM22α‐miR‐485‐3p OE detected by RT‐PCR (*N* = 6). (G) Protein expression levels of NLRP3, BAX/BCL2, and Cleaved caspase3/Caspase3 in AD mouse aortic vascular tissue treated with AAV9‐SM22α‐miR‐485‐3p OE detected by Western blot (*N* = 6). Data between two groups were compared by unpaired two‐tailed Student's *t*‐test. *p < 0.05, **p < 0.01, ***p < 0.001.

Based on the observation that miR‐485‐3p promotes inflammation and apoptosis in HAVSMCs, we hypothesized that VSMC‐specific overexpression of miR‐485‐3p in vivo enhances the development of AD. AAV9‐SM22α‐miR‐485‐3p OE or AAV9‐SM22α‐ctr were tail vein injected into C57BL/6 mice, subsequently followed by the construction of an AD mouse model. Mice in the AAV9‐SM22α‐miR‐485‐3p OE group showed lower survival rates and higher incidence of AD (Figure 3A,B). Simultaneously, in the AAV9‐SM22α‐miR‐485‐3p OE group, the images of the aortic vessels showed a greater degree of intimal tearing (Figure 3C). HE, EVG, and Masson staining indicated a higher degree of vascular lesions and a larger extent of elastic fibre rupture (Figure 3D). The RT‐PCR results demonstrated that compared to the AAV9‐SM22α‐ctr group, the AAV9‐SM22α‐miR‐485‐3p overexpression group exhibited increased expression of miR‐485‐3p in the aortic vessel tissue (Figure 3E), as well as increased expression of inflammatory factors IL1β, IL6 and TNF‐α (Figure 3F). In addition, the results of Western blot analysis showed that the AAV9‐SM22α‐miR‐485‐3p OE group exhibited increased expression of the NLRP3 and an increased ratio of apoptosis proteins BAX/BCL2 and Cleaved caspase3/Caspase3 (Figure 3G). Conclusively, our data indicated that overexpression of VSMC‐specific miR‐485‐3p exacerbates the development of AD in mice by promoting vascular inflammation and cell apoptosis.

### 3.4 VSMC‐specific knockdown of miR‐485‐3p attenuates the development of AD in mice

**FIGURE 4:**
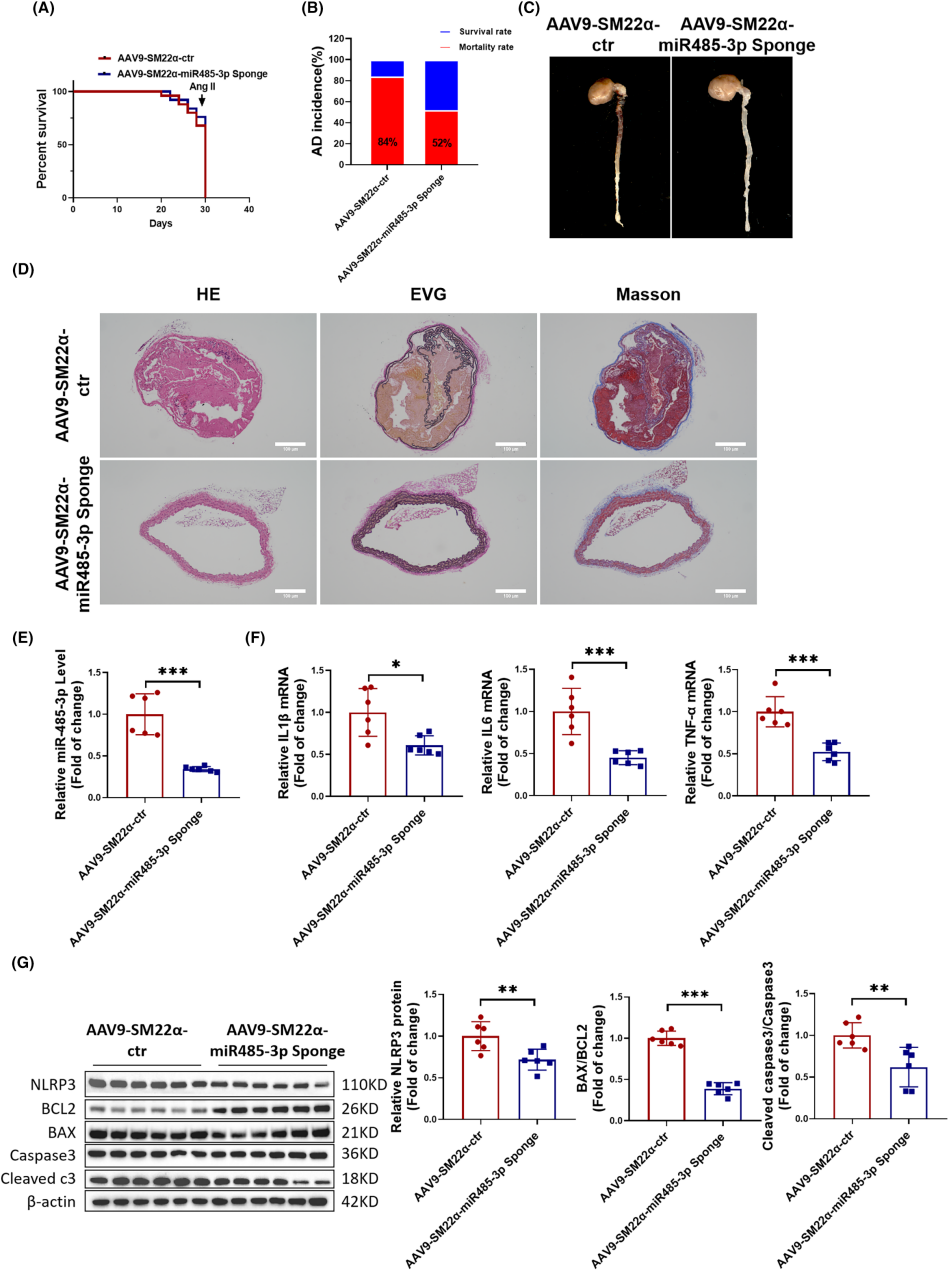
VSMC‐specific knockdown of miR‐485‐3p attenuates the development of aortic dissection (AD) in mice. (A) Mouse survival curve treated with AAV9‐SM22α‐miR‐485‐3p Sponge or AAV9‐SM22α‐ctr followed by AD treatment (*N* = 25). (B) Incidence of AD in mice treated with AAV9‐SM22α‐miR‐485‐3p Sponge (*N* = 25). (C) Images of mouse aortic vascular tissue treated with AAV9‐SM22α‐miR‐485‐3p Sponge. (D) The observation of aortic wall and elastic fibre arrangement in AD mice treated with AAV9‐SM22α‐miR‐485‐3p Sponge using HE staining, EVG staining, and Masson staining, scale bar = 100 μm (*N* = 5). (E) The detection of miR‐485‐3p expression in AD mouse aortas treated with AAV9‐SM22α‐miR‐485‐3p Sponge by RT‐PCR (*N* = 6). (F) RT‐PCR measured mRNA expression levels of inflammatory factors IL1β, IL6 and TNF‐α in AD mouse aortic vascular tissue treated with AAV9‐SM22α‐miR‐485‐3p Sponge (*N* = 6). (G) Western blot detected protein expression levels of NLRP3, BAX/BCL2 and Cleaved caspase3/Caspase3 in AD mouse aortic vascular tissue treated with AAV9‐SM22α‐miR‐485‐3p Sponge (*N* = 6). Data between two groups were compared by unpaired two‐tailed Student's *t*‐test. *p < 0.05, **p < 0.01, ***p < 0.001.

To further investigate the functional role of miR‐485‐3p in vivo, we intravenously injected AAV9‐SM22α‐miR‐485‐3p Sponge or AAV9‐SM22α‐ctr into C57BL/6 mice and established a mouse model of AD. Compared to the AAV9‐SM22α‐ctr group, mice in the AAV9‐SM22α‐miR‐485‐3p Sponge group exhibited a better survival curve and a lower incidence of AD (Figure 4A,B). Similarly, the degree of aortic vascular tear was reduced in the AAV9‐SM22α‐miR‐485‐3p Sponge group (Figure 4C). The tissue staining results also revealed that the degree of vascular lesions and elastic fibre rupture in the AAV9‐SM22α‐miR‐485‐3p Sponge group was reduced (Figure 4D). The RT‐PCR results demonstrated that in the arterial vessel tissues of the AAV9‐SM22α‐miR‐485‐3pSponge group, the expression of miR‐485‐3p was downregulated (Figure 4E), and the secretion of inflammatory factors IL1β, IL6, and TNF‐α was reduced (Figure 4F). Furthermore, Western blot revealed that the expression of NLRP3, BAX/BCL2 and Cleaved caspase3/Caspase3 were reduced (Figure 4G). In brief, our data suggested that knockdown of VSMC‐specific miR‐485‐3p attenuates the development of AD in mice by inhibiting vascular inflammation and cell apoptosis.

### 3.5 miR‐485‐3p targets SIRT1 in HAVSMCs

**FIGURE 5:**
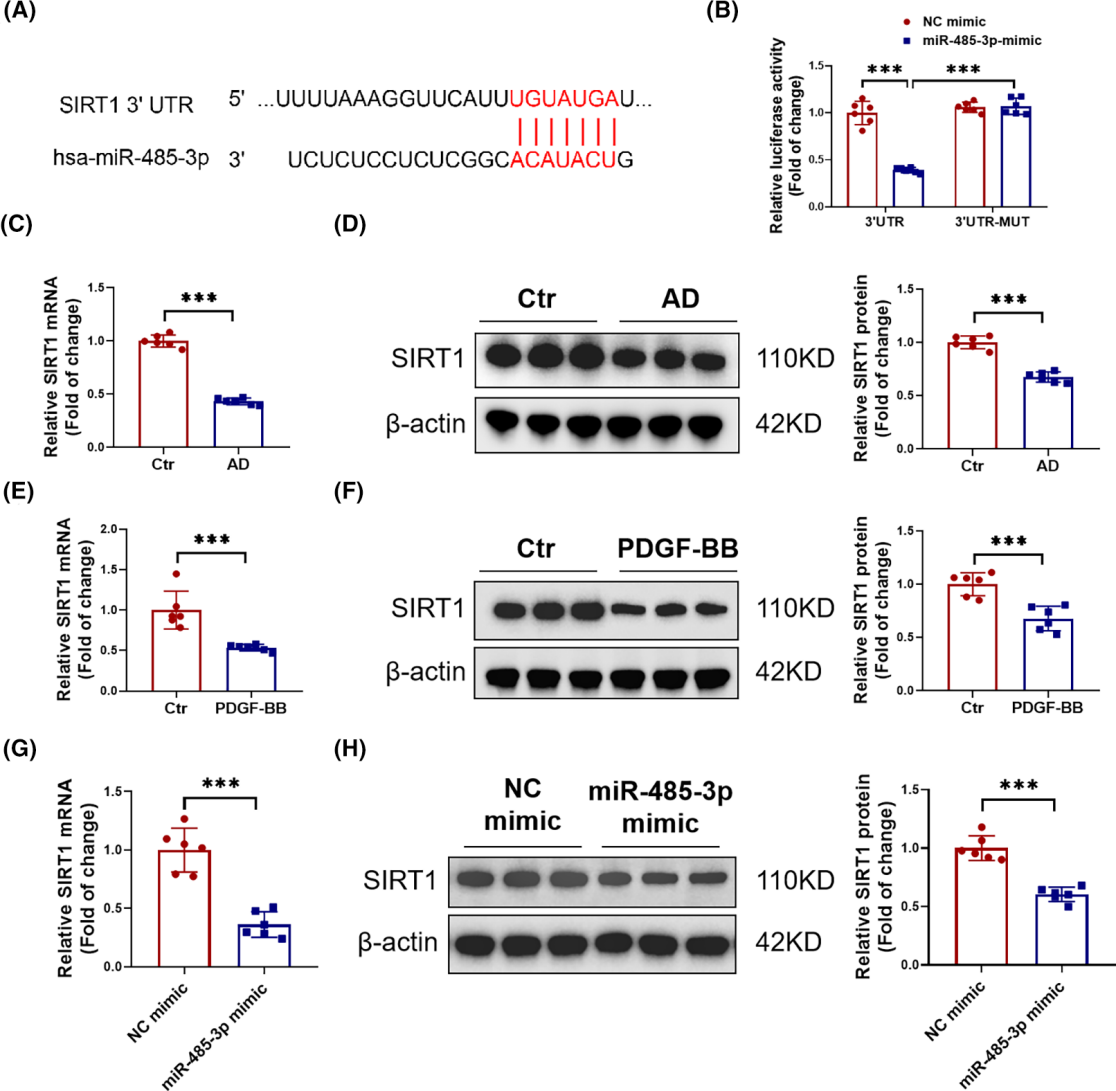
miR‐485‐3p targets SIRT1 in HAVSMCs. (A) Target scan prediction of binding sites between miR‐485‐3p and the 3' UTR of SIRT1. (B) Luciferase reporter assays performed in 293 T cells transfected with miR‐485‐3p mimic (or negative control, NC mimic) and luciferase reporter plasmids containing the binding site in the 3' UTR of SIRT1 or the mutated binding site in the 3' UTR of SIRT1 (*n* = 6). (C–H) The expression of SIRT1 was detected by RT‐PCR and Western blot in AD patients, HAVSMCs treated with PDGF‐BB, and HAVSMCs transfected with miR‐485‐3p mimic (*N* = 6). Data between two groups were compared by unpaired two‐tailed Student's *t*‐test. Data among four groups were compared by two‐way anova followed by Tukey's post hoc test. ***p < 0.001.

In order to identify the target genes of miR‐485‐3p, we performed target gene prediction using TargetScan, and found that SIRT1 has binding sites with miR‐485‐3p (Figure 5A). To confirm whether miR‐485‐3p directly targets SIRT1, we constructed luciferase reporter plasmids containing the binding sequence and mutated sequence in the 3' untranslated region (3' UTR). Co‐transfection with miR‐485‐3p mimic and plasmids containing the binding sequence of SIRT1 3' UTR significantly reduced luciferase activity in 293 T cells, while the reduction of luciferase activity was not present when the 3' UTR binding site was mutated, indicating that miR‐485‐3p could directly target SIRT1 (Figure 5B). Then, in AD patients, we found a decrease in the mRNA and protein levels of SIRT1 (Figure 5C,D). In the in vitro model of AD, a decrease in SIRT1 expression was also observed (Figure 5E,F). In HAVSMCs, we further observed that miR‐485‐3p mimic was effective in downregulating SIRT1 (Figure 5G,H). These results indicated that miR‐485‐3p negatively regulates SIRT1 targeting in HAVSMCs.

### 3.6 SIRT1 deficiency exacerbates AD in vivo

**FIGURE 6:**
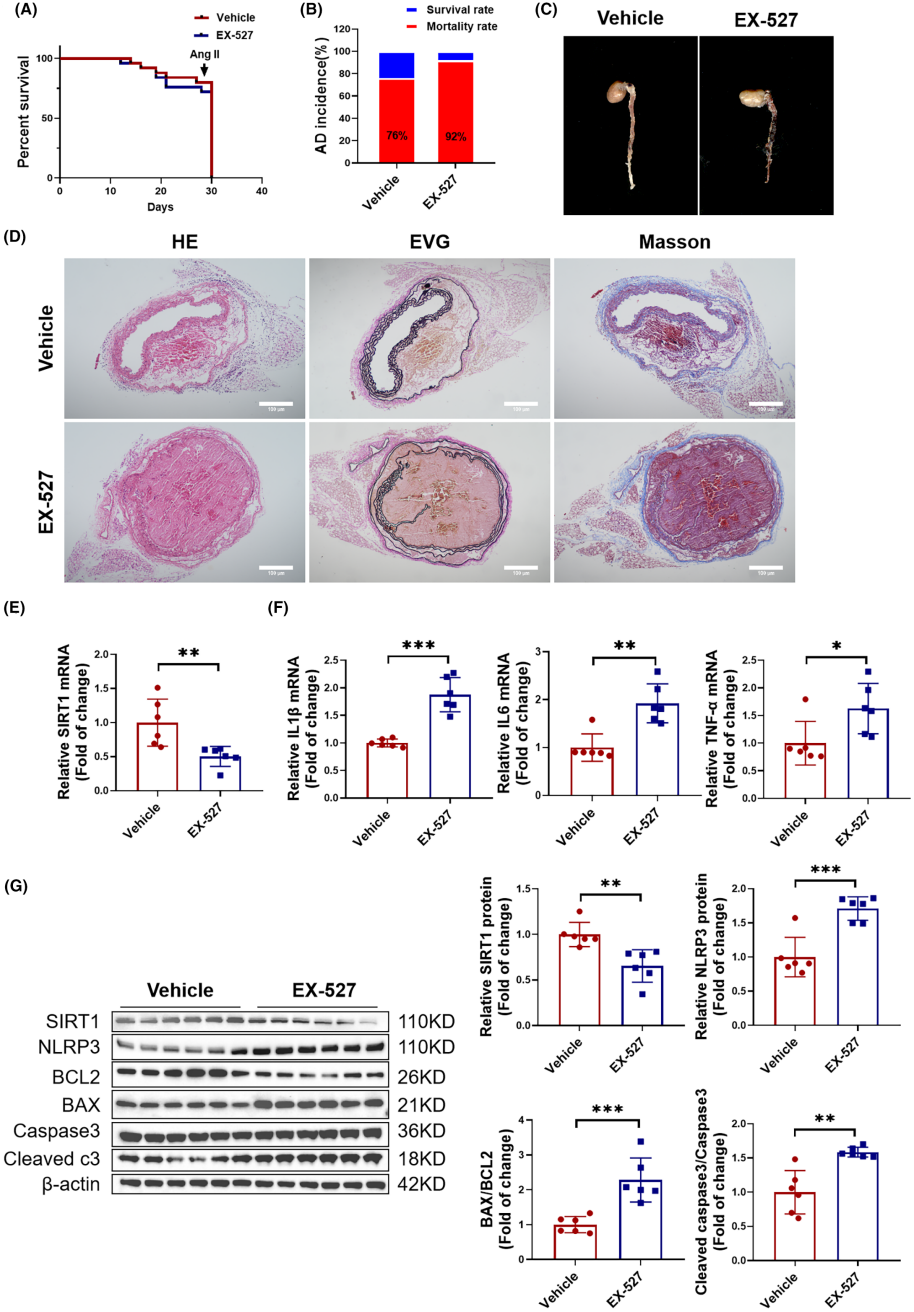
SIRT1 deficiency exacerbates aortic dissection (AD) in vivo. (A) Mouse survival curve treated with EX‐527 or Vehicle followed by AD treatment (*N* = 25). (B) Incidence of AD in mice treated with EX‐527 or Vehicle (*N* = 25). (C) Images of mouse aortic vascular tissue treated with EX‐527 or Vehicle. (D) Observation of aortic wall and elastic fibre arrangement in AD mice treated with EX‐527 or Vehicle using HE staining, EVG staining, and Masson staining, scale bar = 100 μm (*N* = 5). (E) Detection of SIRT1 expression in AD mouse aortas treated with EX‐527 or Vehicle by RT‐PCR (*N* = 6). (F) RT‐PCR tested mRNA expression levels of inflammatory factors IL1β, IL6 and TNF‐α in AD mouse aortic vascular tissue treated with EX‐527 or Vehicle (*N* = 6). (G) Western blot measured Protein expression levels of SIRT1, NLRP3, BAX/BCL2 and Cleaved caspase3/Caspase3 in AD mouse aortic vascular tissue treated with EX‐527 or Vehicle (*N* = 6). Data between two groups were compared by unpaired two‐tailed Student's *t*‐test. *p < 0.05, **p < 0.01, ***p < 0.001.

To elucidate the role of SIRT1 in AD, we orally administered the SIRT1 inhibitor EX‐527 to four‐week‐old mice and then established an AD model. The results revealed that mice with suppressed SIRT1 expression exhibited poorer survival rates and an increased incidence of AD (Figure 6A,B). Furthermore, imaging of the aortic blood vessels showed a higher degree of intimal tear in the EX‐527 group (Figure 6C), and tissue staining indicated a higher degree of vascular pathology and greater elastin fibre rupture in the EX‐527 group (Figure 6D). Additionally, RT‐PCR analysis revealed a decrease in the expression of SIRT1 in the aortic vessel tissue of the EX‐527 group (Figure 6E), accompanied by an increase in the expression of inflammatory factors IL1β, IL6 and TNF‐α (Figure 6F). The Western blot analysis results also indicated that the expression of SIRT1 was decreased, while the expression of NLRP3, BAX/BCL2 and Cleaved caspase3/Caspase3 was increased in the EX‐527 group mice (Figure 6G). In short, our results indicated that the inhibition of SIRT1 exacerbates vascular inflammation and apoptosis, thereby promoting the development of AD in mice.

### 3.7 SIRT 1 activation alleviates AD in vivo

**FIGURE 7:**
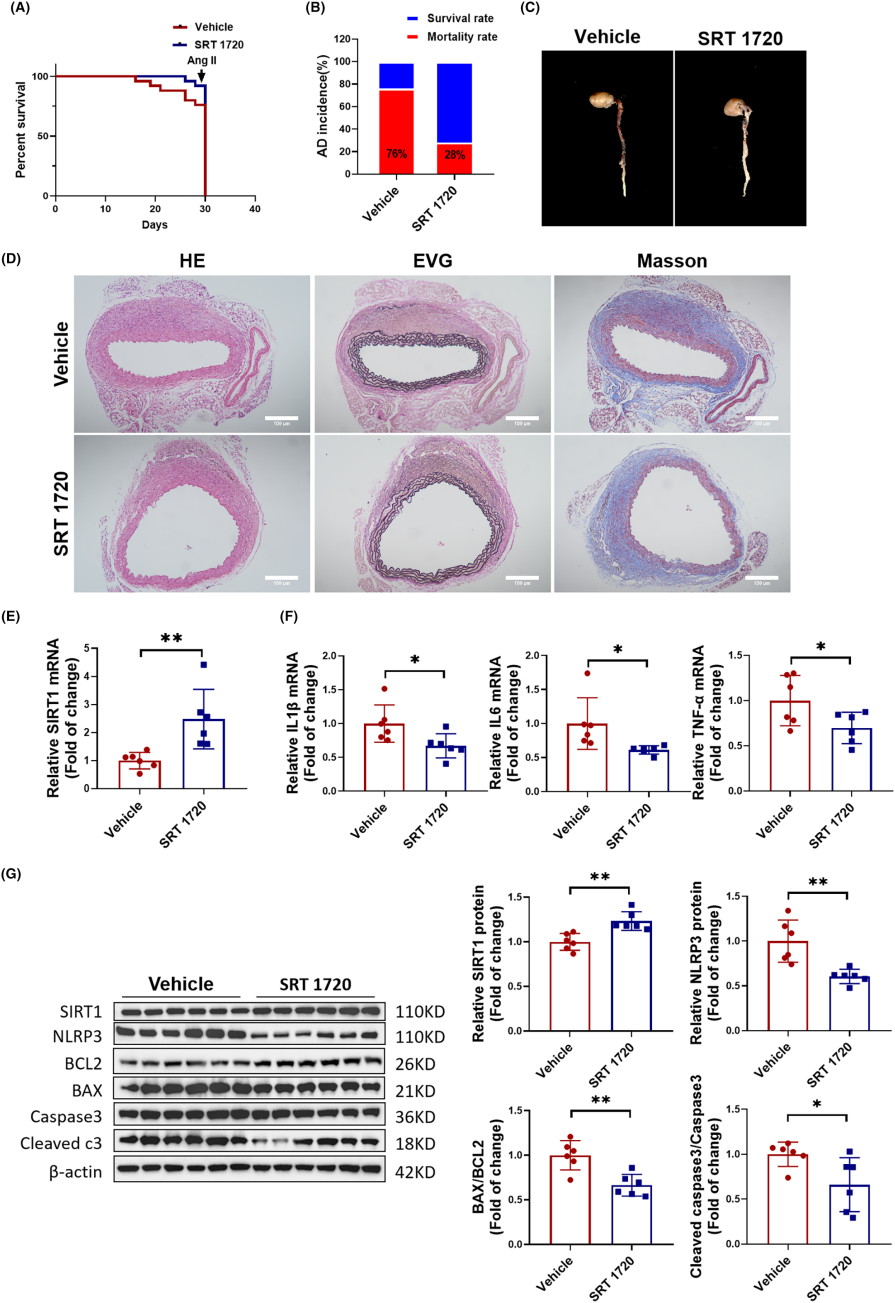
SIRT1 activation alleviates aortic dissection (AD) in vivo. (A) Mouse survival curve treated with SRT 1720 or Vehicle followed by AD treatment (*N* = 25). (B) Incidence of AD in mice treated with SRT 1720 or Vehicle (*N* = 25). (C) Images of mouse aortic vascular tissue treated with SRT 1720 or Vehicle. (D) Observation of aortic wall and elastic fibre arrangement in AD mice treated with SRT 1720 or Vehicle using HE staining, EVG staining and Masson staining, scale bar = 100 μm (*N* = 5). (E) Detection of SIRT1 expression in AD mouse aortas treated with SRT 1720 or Vehicle by RT‐PCR (*N* = 6). (F) RT‐PCR for mRNA expression levels of inflammatory factors IL1β, IL6, and TNF‐α in AD mouse aortic vascular tissue treated with SRT 1720 or Vehicle (*N* = 6). (G) Western blot for protein expression levels of SIRT1, NLRP3, BAX/BCL2 and Cleaved caspase3/Caspase3 in AD mouse aortic vascular tissue treated with SRT 1720 or Vehicle (*N* = 6). Data between two groups were compared by unpaired two‐tailed Student's *t*‐test. *p < 0.05, **p < 0.01.

To investigate the effect of SIRT 1 activation on the development of AD in mice, we orally administered the SIRT1 agonist SRT 1720 to mice and induced the AD model, which lasted for 4 weeks. We found that the mice had better survival curves and significantly lower dissection rates in the SRT 1720 group (Figure 7A,B). The aortic vascular images also demonstrated a reduced degree of vascular tears in the SRT 1720 group (Figure 7C). HE, EVG and Masson staining further revealed fewer vascular lesions and elastic fibre ruptures in the SRT 1720 group (Figure 7D). Subsequently, we found an increased expression of SIRT1 (Figure 7E), accompanied by a decreased expression of the inflammatory factors IL1β, IL6 and TNF‐α (Figure 7F). Additionally, Western blot results also showed an increased protein expression of SIRT1 and a decreased expression of NLRP3, BAX/BCL2 and Cleaved caspase3/Caspase3 in the aortic vascular tissues of the SRT 1720 group mice (Figure 7G). Based on these findings, we concluded that the activation of SIRT1 can inhibit vascular inflammation and apoptosis, thereby suppressing the development of AD in mice.

### 3.8 SIRT1 reverses the inflammation and apoptosis induced by miR‐485‐3p in HAVSMCs

**FIGURE 8:**
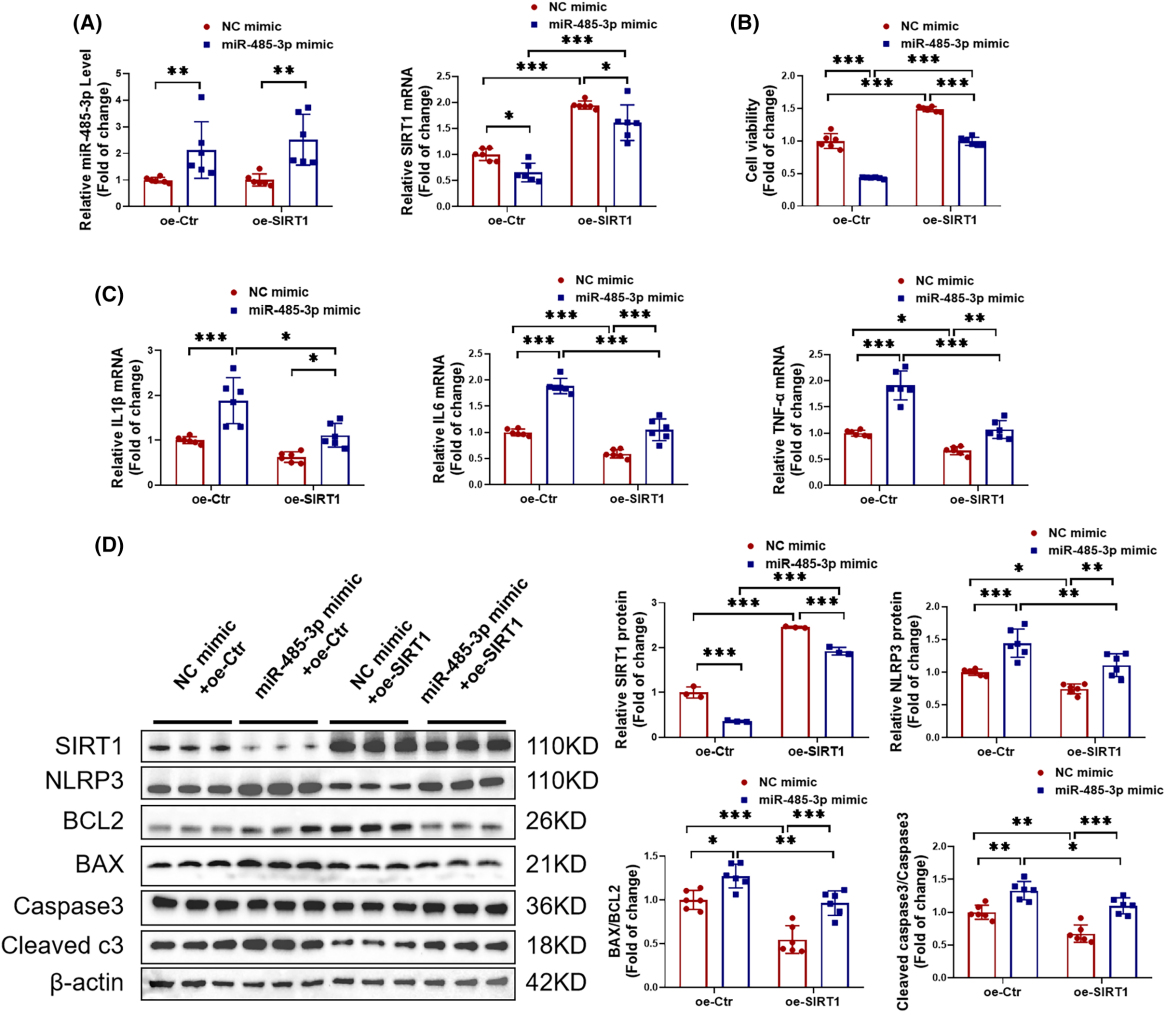
SIRT1 reverses the inflammation and apoptosis induced by miR‐485‐3p in HAVSMCs. HAVSMCs were co‐transfected with miR‐485‐3p mimic and SIRT1 overexpression plasmid, and then treated with PDGF‐BB. (A) RT‐PCR detected the expression of miR‐485‐3p and SIRT1 (*N* = 6). (B) CCK8 assay assessed the cell viability (*N* = 6). (C) RT‐PCR detected the expression of inflammatory cytokines IL1β, IL6 and TNF‐α (*N* = 6). (D) Western blot analysis determined the expression of SIRT1, NLRP3, BAX/BCL2 and Cleaved caspase3/Caspase3 proteins (*N* = 3–6). Data among four groups were compared by two‐way anova followed by Tukey's post hoc test. *p < 0.05, **p < 0.01, ***p < 0.001.

In order to investigate the interaction between miR‐485‐3p and SIRT1, we co‐transfected miR‐485‐3p mimic and SIRT1 overexpression plasmid into HAVSMCs and then treated them with PDGF‐BB to establish an in vitro AD model. First, the overexpression efficiency was determined by RT‐PCR, showing an increase in the expression of miR‐485‐3p and SIRT1 (Figure 8A). CCK‐8 assay revealed that overexpression of SIRT1 alleviated the decrease in cell viability induced by PDGF‐BB in HAVSMCs and further reversed the decrease in cell viability mediated by miR‐485‐3p (Figure 8B). Additionally, increased expression of SIRT1 reversed the upregulation of pro‐inflammatory factors IL1β, IL6 and TNF‐α mediated by miR‐485‐3p (Figure 8C). Protein levels of NLRP3, BAX/BCL2 and Cleaved caspase3/Caspase3 were also decreased (Figure 8D). Similarly, we co‐transfected miR‐485‐3p mimic and SIRT1 knockdown plasmid into HAVSMCs and treated them with PDGF‐BB. The results indicated that overexpression of miR‐485‐3p and knockdown of SIRT1 both promote inflammation and apoptosis in HAVSMCs (Figure S4). In summary, miR‐485‐3p negatively regulates SIRT1 to promote inflammation and apoptosis in HAVSMCs.

## 4 DISCUSSION

It is crucial to elucidate the potential mechanisms and identify effective therapeutic targets for AD, a cardiovascular disease associated with high mortality rates.^22^ MiRNAs can participate in the occurrence and development of AD through various pathways.^14^, ^23^ However, current research in this field is still in its early stages, and the exact mechanism of how miRNA functions in AD, as well as how to translate these understandings into therapeutic approaches, remains to be unveiled through further in‐depth studies. In this study, we observed a significant upregulation of miR‐485‐3p in human AD tissues as well as in HAVSMCs treated with PDGF‐BB.

miR‐485‐3p is involved in various cellular processes such as inflammation, migration, proliferation and apoptosis, which are closely associated with diseases.^12^, ^24^, ^25^, ^26^ In atherosclerosis, miR‐485‐3p targets Olr1 to regulate vascular inflammation and lipid transport, thereby participating in the development of the disease.^14^ In paediatric asthma, miR‐485‐3p can also promote inflammatory response and extracellular matrix deposition by activating the Wnt/β‐catenin signalling pathway in human airway smooth muscle cells.^27^ To uncover the function of miR‐485‐3p in AD, we conducted extensive research. At the cellular level, we found that overexpression of miR‐485‐3p in HAVSMCs led to heightened inflammation and increased apoptosis, while downregulation of miR‐485‐3p suppressed these processes. Moving to in vivo experiments using animal models, we discovered that specific overexpression of miR‐485‐3p in VSMCs promoted vascular inflammation and tissue apoptosis in AD mice, concurrently increasing the disease incidence and mouse mortality. Conversely, when we specifically knocked down miR‐485‐3p in VSMCs, we observed reduced vascular inflammation and apoptosis in AD mice, accompanied by a decrease in AD occurrence and death rate. These comprehensive findings strongly suggest that miR‐485‐3p plays a crucial role in the development of AD by regulating the inflammatory response and apoptosis of smooth muscle cells. This discovery offers a new perspective for understanding and potentially treating AD, identifying miR‐485‐3p as a potential therapeutic target.

After confirming that miR‐485‐3p can regulate inflammation and apoptosis in AD, we further investigated its molecular mechanisms. Through bioinformatics analysis, it was found that miR‐485‐3p can target SIRT1. SIRT1 has been widely recognized to be involved in the regulation of cellular metabolism, oxidative stress, inflammation, and apoptosis, all of which contribute to the development of cardiovascular diseases.^28^, ^29^, ^30^ For example, SIRT1 can inhibit the regulation of vascular inflammation by NF‐κB, TNF‐α and the NLRP3 inflammasome.^31^, ^32^ SIRT1 also regulates vascular cell apoptosis by targeting cell death‐related proteins such as p53 and FoxO.^33^, ^34^ Our research indicated that SIRT1, as a crucial downstream target of miR‐485‐3p, is closely associated with the pathogenesis of AD. In in vivo experiments, we observed that the inhibition of SIRT1 exacerbates vascular inflammation and tissue apoptosis, thus promoting the formation of AD. Conversely, the activation of SIRT1 significantly reduces these adverse effects, effectively alleviating the progression of AD. Through reversal function Experiments, we confirmed that activating SIRT1 can counteract the inflammation and apoptosis in HAVSMCs induced by miR‐485‐3p. Therefore, we concluded that SIRT1 is a key direct target through which miR‐485‐3p regulates vascular inflammation and apoptosis, thereby influencing the occurrence of AD.

Currently, our study has several limitations. Firstly, we have yet to successfully construct gene‐edited mice specifically overexpressing or knocking down miR‐485‐3p in VSMCs to further observe the role of miR‐485‐3p in the development of AD. Secondly, our study is limited to only one model of AD. Future research should consider employing multiple models to gain a more comprehensive understanding of miR‐485‐3p's function in AD. Lastly, while we have identified SIRT1 as a downstream target of miR‐485‐3p, contributing to the regulation of AD, there may be other undiscovered downstream targets of miR‐485‐3p that could play crucial roles in the pathological process of AD. Despite these limitations, this study revealed new mechanisms underlying AD, and the miR‐485‐3p/SIRT1 may serve as a novel therapeutic target for AD.

In summary, our findings indicated that miR‐485‐3p regulates the inflammatory response and apoptosis in HAVSMCs by suppressing SIRT1 expression, thus mediating the development of AD. Inhibiting miR‐485‐3p or promoting the expression of SIRT1 can alleviate vascular inflammation and cell apoptosis, thereby alleviating the progression of AD.

## AUTHOR CONTRIBUTIONS

**Yuling Xie:** Conceptualization (equal); data curation (equal); writing – original draft (lead). **Linfeng Xie:** Conceptualization (equal); data curation (equal). **Zhihuang Qiu:** Data curation (equal); formal analysis (equal). **Jian He:** Data curation (equal); formal analysis (equal). **Fei Jiang:** Formal analysis (equal). **Meiling Cai:** Methodology (equal). **Yanjuan Lin:** Conceptualization (equal); funding acquisition (lead); writing – review and editing (lead). **Liangwan Chen:** Conceptualization (equal); data curation (equal); funding acquisition (lead); writing – review and editing (lead).

## FUNDING INFORMATION

This work was supported by Fujian Provincial Special Reserve Talents (2021‐25); the National Natural Science Foundation of China (U2005202), (82370470) and (82241209); and Startup Fund for scientific research (Fujian Medical University, Grant number: 2023QH1034).

## CONFLICT OF INTEREST STATEMENT

The authors confirm that there are no conflicts of interest.

## Supporting information


Data S1.

## Data Availability

The data used to support the findings of this study are available from the corresponding author upon reasonable request.
