# Supplementary material for: miR‐485‐3p targets SIRT1 in vascular smooth muscle cells mediating the occurrence of aortic dissection
Source: J Cell Mol Med. 2024 Jul 15;28(13):e18454. doi: 10.1111/jcmm.18454 (PMC11250145; doi:10.1111/jcmm.18454)
Supplement: Supplementary file 1 — Data S1. [file JCMM-28-e18454-s001.docx]

**Supplemental material**

**Table S1 The demographic and clinical characteristics of the included AD patients and controls.**

|  | AD （n=12） | Ctr （n=12） | P value |
| --- | --- | --- | --- |
| Age, years | 54.9 ± 8.6 | 51.9 ± 7.6 | 0.397 |
| Sex, male:female | 12:00 | 12:00 | 0.999 |
| Hypertension, n(%) | 8(66.6%) | 2(16.6%) | 0.036 |
| Hyperlipidemia, n(%) | 4(33.3%) | 0(0.0%) | 0.0932 |
| Diabetes mellitus, n(%) | 1(8.3%) | 0(0.0%) | 0.999 |
| Coronary artery disease, n(%) | 2(16.6%) | 0(0%) | 0.4783 |
| Infectious disease, n(%) | 0(0%) | 0(0%) | 0.999 |
| Trauma history, n(%) | 0(0%) | 0(0%) | 0.999 |
| Operation history, n(%) | 0(0%) | 0(0%) | 0.999 |
| Transfusion history, n(%) | 0(0%) | 0(0%) | 0.999 |
| Allergy history, n(%) | 0(0%) | 0(0%) | 0.999 |
| Smoking history, n(%) | 6(50.0%) | 4(33.3%) | 0.6802 |
| Drinking history, n(%) | 7(58.3%) | 6(50%) | 0.999 |
| Stanford classification | Stanford A | N/A | N/A |

**Table S2 Primer sequences.**

| Gene | Forward | Reserve |
| --- | --- | --- |
| hsa-IL1β | GCTTATTACAGTGGCAATGAGGAT | TAGTGGTGGTCGGAGATTCG |
| hsa-IL6 | ATGCAATAACCACCCCTGAC | GCGCAGAATGAGATGAGTTGT |
| hsa-TNF-α | TCCTCTCTGCCATCAAGAGC | AGTAGACCTGCCCAGACTCG |
| hsa-SIRT1 | TATACCCAGAACATAGACACGC | CTCTGGTTTCATGATAGCAAGC |
| hsa-β-actin | GAGAAAATCTGGCACCACACC | GGATAGCACAGCCTGGATAGCAA |
| hsa-SM22α | AGAATGATGGGCACTACCGTG | CTGTTGCTGCCCATCTGAAG |
| hsa-SMA | CCTGACTGAGCGTGGCTATT | GCCCATCAGGCAACTCGTAA |
| hsa-PCNA | CACTCCACTCTCTTCAACGGT | ATCCTCGATCTTGGGAGCCA |
| mus-IL1β | GAAATGCCACCTTTTGACAGTG | TGGATGCTCTCATCAGGACAG |
| mus-IL6 | CTGCAAGAGACTTCCATCCAG | AGTGGTATAGACAGGTCTGTTGG |
| mus-TNF-α | GCCGATGGGTTGTACCTTGT | TCTTGACGGCAGAGAGGAGG |
| mus-SIRT1 | ACCACCAAATCGTTACATATTCCA | CCGTATCATCTTCCAAGCCATT |
| mus-β-actin | TGTCCACCTTCCAGCAGATGT | AGCTCAGTAACAGTCCGCCTAG |
| mus-SM22α | GAAGGTGCCTGAGAACCCAC | TGCTGCCATATCCTTACCTTCA |
| mus-SMA | GCATCCACGAAACCACCTATAAC | ACAGAGTACTTGCGTTCTGGAG |
| mus-PCNA | TATGCCGAGACCTTAGCCAC | TCTCTATGGTTACCGCCTCCT |


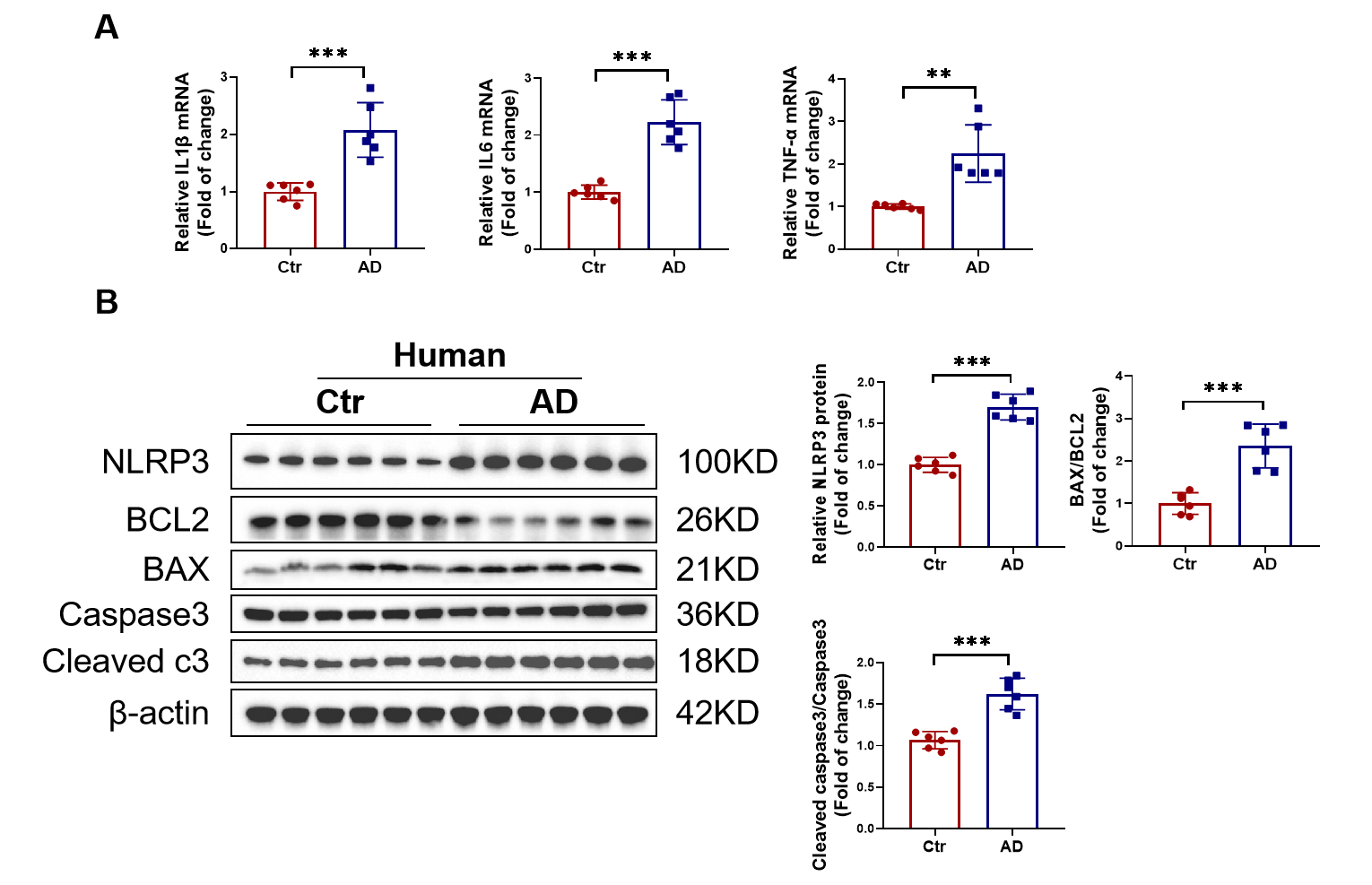


**Figure S1 Increased inflammation and apoptosis in the aortic dissection group.** (A) RT-PCR determined the expression levels of inflammatory factors IL1β, IL6 and TNF-α in the aortic dissection (AD) group and the healthy control group (N=6). (B) Western blot analysis detected the expression of NLRP3, BAX/BCL2 and Cleaved caspase3/Caspase3 proteins in the AD group and the healthy control group (N=6). Data between two groups were compared by unpaired two-tailed Student's t test. ∗∗p < 0.01, ∗∗∗p < 0.001.


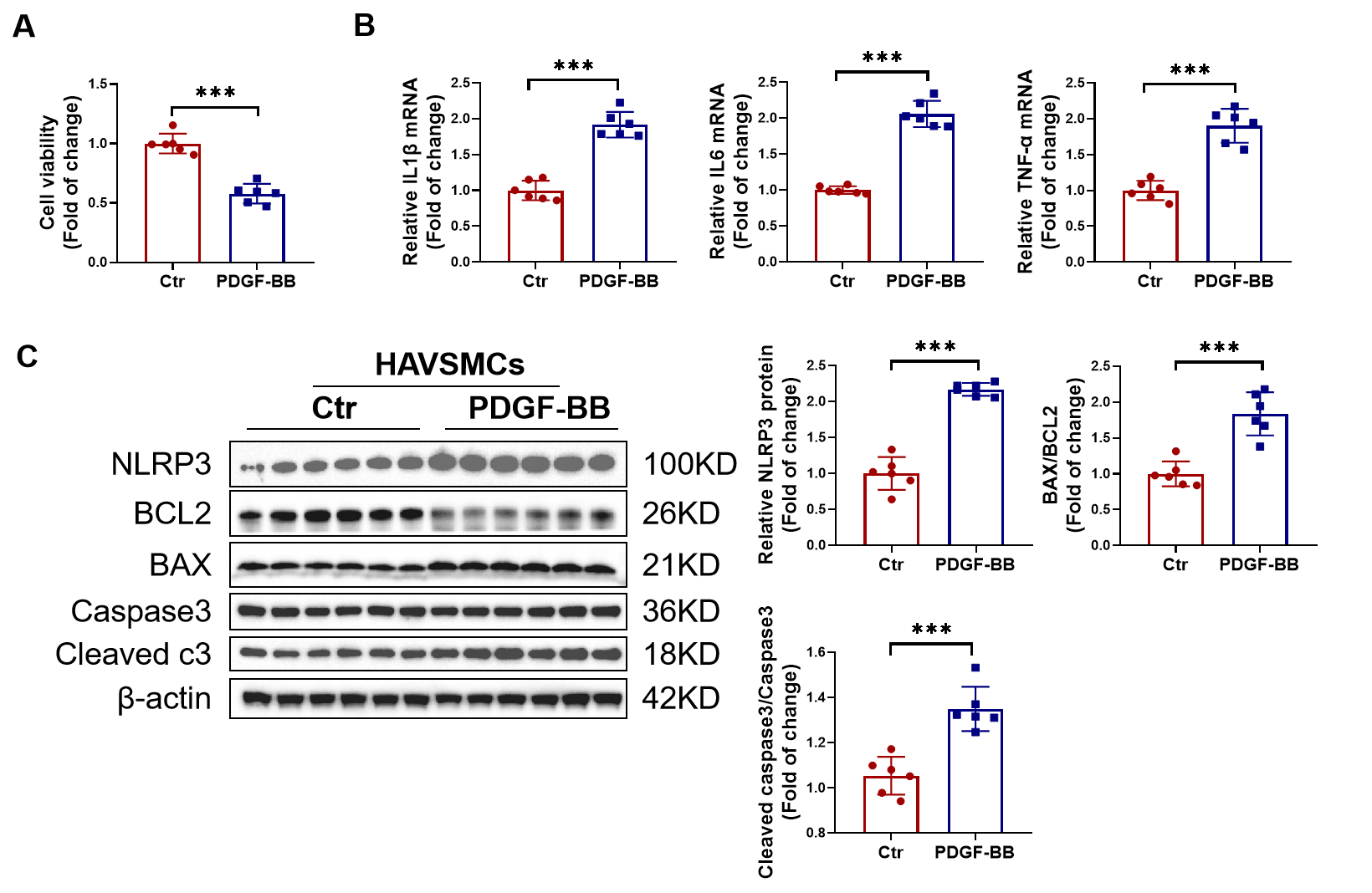


**Figure S2 PDGF-BB induces inflammation and apoptosis in HAVSMCs.** (A) CCK8 assay assessed the cell viability in PDGF-BB-treated HAVSMCs (N=6). (B) RT-PCR determined the expression levels of inflammatory factors IL1β, IL6 and TNF-α in PDGF-BB-treated HAVSMCs (N=6). (C) Western blot detected the expression of NLRP3, BAX/BCL2 and Cleaved caspase3/Caspase3 proteins in PDGF-BB-treated HAVSMCs (N=6). Data between two groups were compared by unpaired two-tailed Student's t test. ∗∗∗p < 0.001.


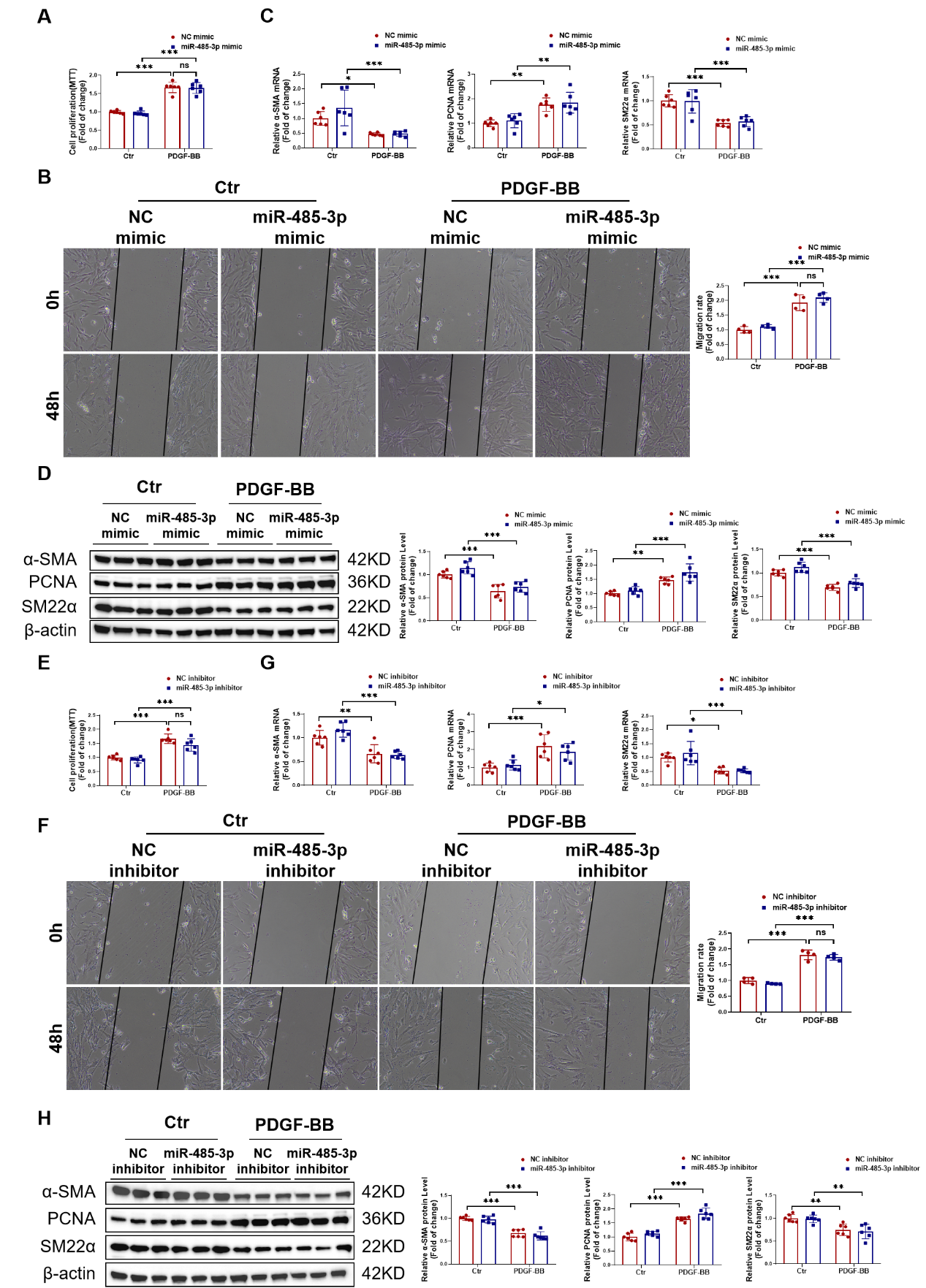


**Figure S3 miR-485-3p does not regulate the proliferation, migration, and phenotypic transformation of HAVSMCs.** (A-D) HAVSMCs were transfected with miR-485-3p mimic and then treated with PDGF-BB. (A) MTT assay for cell proliferation detection (N=6). (B) Wound scratch assay for cell migration detection (N=6). (C-D) RT-PCR and Western blot determined the expression levels α-SMA, PCNA and SM22α (N=6). (E-H) HAVSMCs were transfected with miR-485-3p inhibitor and then treated with PDGF-BB. (E) MTT assay for cell proliferation detection (N=6). (F) Wound scratch assay for cell migration detection (N=4). (G-H) RT-PCR and Western blot detected the expression of α-SMA, PCNA and SM22α (N=6). Data among four groups were compared by two-way ANOVA followed by Tukey’s *post hoc* test. ∗p < 0.05, ∗∗p < 0.01, ∗∗∗p < 0.001.


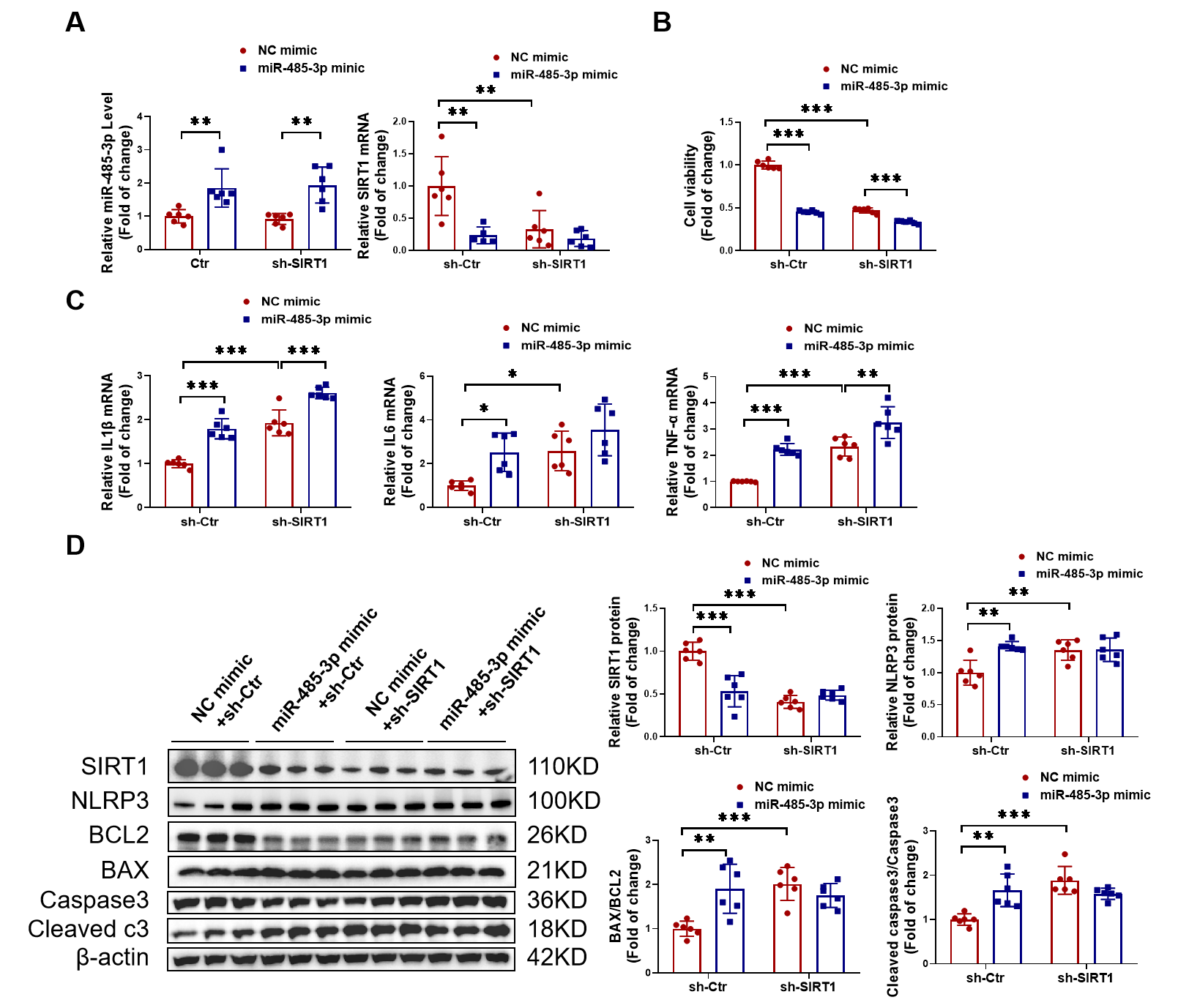


**Figure S4 SIRT1 knockdown and miR-485-3p overexpression similarly promote inflammation and apoptosis in HAVSMCs.** HAVSMCs were co-transfected with miR-485-3p mimic and SIRT1 knockdown plasmid, and treated with PDGF-BB. (A) RT-PCR detected the expression of miR-485-3p and SIRT1 (N=6). (B) CCK8 assay assessed the cell viability (N=6). (C) RT-PCR detected the expression of inflammatory cytokines IL1β, IL6, and TNF-α (N=6). (D) Western blot analysis determined the expression of SIRT1, NLRP3, BAX/BCL2 and Cleaved caspase3/Caspase3 proteins (N=6). Data among four groups were compared by two-way ANOVA followed by Tukey’s *post hoc* test. ∗p < 0.05, ∗∗p < 0.01, ∗∗∗p < 0.001.
